# Acknowledgment to Reviewers of *Diseases* in 2021

**DOI:** 10.3390/diseases10010008

**Published:** 2022-01-27

**Authors:** Diseases Editorial Office

**Affiliations:** MDPI AG, St. Alban-Anlage 66, 4052 Basel, Switzerland

Rigorous peer-reviews are the basis of high-quality academic publishing. Thanks to the great efforts of our reviewers, *Diseases* was able to maintain its standards for the high quality of its published papers. Thanks to the contribution of our reviewers, in 2021, the median time to first decision was 21 days, and the median time to publication was 43.5 days. The editors would like to extend their gratitude and recognition to the following reviewers for their precious time and dedication, regardless of whether the papers they reviewed were finally published:
Abdeen, AhmedLichtenauer, MichaelAgüero-Chapin, GuillerminLima, MarceloAhmed, HebaLisotti, AndreaAlcántara-Ortigoza, Miguel AngelLiu, Chung-JungAlfie, JoséLoretz, BrigittaAmero, CarlosLucke-Wold, BrandonAndreoni, FrancescaLuigi, LavorgnaArenas, RobertoMacaluso, GiusiArmesto Alonso, SusanaMagrassi, LorenzoArrivillaga, JazzminMallmann, Michael RudolfAtanasova, KalinaMani, AlirezaAvin, VijayMathew, RajammaBaldi, IleanaMathieu, DavidBallestri, StefanoMauri, DavideBalogh, EnikőMeggyes, MátyásBao, XiucongMeng, YanBarnea, ItayMonroy, FernandoBehringer, ErikMoon, Jee-YoungBelosludtseva, Natalia V.Moreira, Mário W. L.Bel’skaya, LyudmilaMorford, LorriBenítez Sillero, Juan De DiosNaldi, LuigiBennasar, MarOftadeh, ShahinBojarska-Junak, Agnieszka A.Ogino, ShujiBrady, GarethOlschewski, HorstBrasic, JamesOwczarczyk-Saczonek, AgnieszkaBrooke, JoannePanzuto, FrancescoBrown, GregoryParadkar, PrasadBrown, Ronald B.Parkar, Shanthi G.Buckingham, LelaPeh, Hong YongCaccamo, DanielaPeitsidis, PanagiotisCai, QiangjunPellegrinelli, LauraCăinap, CălinPeres, Nalu Teixeira AguiarCanales, JimenaPeserico, AlessiaCarlisi, DanielaPeters, John C.Carta, AngelaPicó-Monllor, José AntonioChela, HarleenPieper, BarbaraCho, Joo-YounPintos-Morell, GuillemCoimbra, Ibsen B.Pogreba-Brown, KristenCorea, FrancescoPolovic, NatalijaCosta, SolangePontes, BrunoCosta-Bauzá, AntòniaPop, DanaCsaderova, LuciaPrats, Anne-CatherineCurrò, MonicaPripp, Are HugoDavid, González-BarrioPyza, ElżbietaDavis, David A.Rangel, Érika B.Di Marco, RobertoRazvan-Cosmin, PetcaDuc, Nguyen MinhReyes-Ortega, FelisaEl-Sheikh Ali, HossamRichardson, Sandra R.Espinosa, EmmaRocchiccioli, SilviaEsteban, Luis MarianoRomani, PatriziaFatone, GerardoRossi, AlessandroFenoglio, RobertaRoussakow, Sergey V.Ferreira, NelsonRoutledge, MichaelFiering, Steve N.Ruan, KeFife-Schaw, ChrisRusso, AntonioFranchini, MassimoSalucci, SaraFranco, Roberto S.Sanchez, Maria T.Frías-De-León, María GuadalupeSarid, OrlyFulmer, Brant R.Sato, EiichiGamarra, Lionel FernelSerre, KarineGarinis, Angela C.Shen, Ming-YiGatzoulis, Konstantinos A.Shoshkes-Carmel, MichalGeorge, AndersonSiqueira, Hugo ValadaresGhosh, Arjun KumarSivula, MirkaGodman, BrianSmaga, IrenaGómez-Barrena, EnriqueSong, Mi-YeonGozes, IllanaSoreq, HermonaGranata, AntonioSpennato, PietroHaque, MuhammadStabile, GuglielmoHeijman, JordiStellas, DimitrisHernández-Vara, JorgeStricker, HansHildreth, BlakeStridh, PernillaHong, GuojuSubbiah, MuruganHorng, Chi-TingSzeliga, MonikaHorowitz, John D.Tabachnik Schor, Nina F.Horowitz, Robert A.Takahashi, TetsufumiHouser, Madelyn C.Takala, JukkaHuang, FangTaradaj, JakubJardini, Maria Aparecida NevesTéllez-Valencia, AlfredoJiang, Jhih-HangTesarik, JanJohanning, EckardtToietta, GabrieleJovanov-Milošević, NatašaTonhajzerova, IngridJung, Ji HoonUzunoglu, Faik G.Kala, RishabhVafiadaki, ElizabethKang, JeonghyunVandenbroeck, KoenKanno, TakeshiVeranic, PeterKano, SatoshiViana, Joao Henrique MoreiraKidd, Lisa A.Wang, HsiuyingKleczyński, PawełWang, KaiKobeissy, FirasWennmann, JörgKoyama, SachikoWiedmann, FelixKrams, IndrikisWróbel, AgnieszkaKrela-Kaźmierczak, IwonaWu, Meng-YuKucukseymen, SelcukXu, Yan-MingKuliczkowski, KazimierzYatomi, MasakiyoLasithiotakis, KonstantinosYeligar, Samantha M.Lee, Chii-MingYen, Cheng-FangLeng, GarethZaman, ShafquatLi, MingqiangZarobkiewicz, MichałLi, SunanZingg, Jean-Marc


